# Body mass index and waist circumference trajectories across the life course and birth cohorts, 1996–2015 Malaysia: sex and ethnicity matter

**DOI:** 10.1038/s41366-023-01391-5

**Published:** 2023-10-13

**Authors:** Chien Huey Teh, Sanjay Rampal, Chee Cheong Kee, Omar Azahadi, Aris Tahir

**Affiliations:** 1 Centre for Epidemiology and Evidence-based Practice, Department of Social and Preventive Medicine, Faculty of Medicine, University of Malaya, 50603 Kuala Lumpur, Malaysia; 2 Institute for Medical Research, National Institutes of Health, Ministry of Health Malaysia, 40170 Setia Alam, Malaysia; 3 Sector for Biostatistics and Data Repository, National Institutes of Health, Ministry of Health Malaysia, 40170 Setia Alam, Malaysia

**Keywords:** Risk factors, Obesity, Epidemiology

## Abstract

**Objective:**

The global obesity epidemic remains a significant threat to public health and the economy. Age-period-cohort (APC) analysis is one method to model the trajectory of obesity. However, there is scarce published evidence of such analyses among the South East Asian population. This study aims to explore the sex and ethnic variations of BMI and waist circumference trajectories over time among non-institutionalized Malaysian adults aged 18 to 80 years.

**Methods:**

Data from four population-based National Health and Morbidity Surveys conducted in 1996, 2006, 2010, and 2015 were pooled. Hierarchical Age-Period-Cohort (HAPC) analysis explored the trajectories of BMI and waist circumference across the life course and birth cohorts by sex and ethnicity. These models assumed no period effect.

**Results:**

Generally, BMI and waist circumference trajectories increased across age and birth cohorts. These trajectories varied by sex and ethnicity. Females have more profound increasing BMI and waist circumference trajectories than their male counterparts as they age and as cohort recency increases. Chinese have less profound BMI and waist circumference increases across the life course and birth cohorts than other ethnic groups.

**Conclusions:**

The profound increasing cohort trajectories of obesity, regardless of sex and ethnicity, are alarming. Future studies should focus on identifying factors associated with the less profound cohort effect among the Chinese to reduce the magnitude of trajectories in obesity, particularly among future generations.

## Introduction

Obesity is a significant public health threat. It is a major risk factor for noncommunicable diseases such as cardiovascular diseases, diabetes, and certain cancers [1]. Obesity also increases the risk of COVID-19 morbidity and mortality [2]. Globally, obesity was associated with an estimated 160 million disability-adjusted life years (DALYs) and 5.0 million premature deaths per annum in 2019 [3].

Obesity has been increasingly prevalent in Malaysia since 1996 [4] and has become more common among younger age groups [5]. However, previous trend analyses compared point estimates from different surveys without considering the mixed effects of age, period, and cohort (APC). Ignoring these three effects may introduce confounding. For instance, the positive age-BMI associations reported in cross-sectional studies may be confounded by intrapersonal ageing-related changes (age effect), secular changes that affect everyone regardless of age and birth year (period effect), or sociodemographic changes in the population due to unique differences in environmental, societal and historical exposure between people born in different cohorts (cohort effect).

More recent cohorts may have increasingly obesogenic environments due to increased consumption of energy-densel and nutrient-dilute diets. Changes in the built environment and reduced active transportation over time may also decrease physical activity. It is thus important to account for inter-cohort (i.e., cohort effects) and intra-cohort variations due to sex and ethnic differences when modeling BMI and waist circumference trajectories, particularly in a rapidly evolving, multi-ethnic country like Malaysia.

Evidence on the associations between sex and BMI and waist circumference trajectories has not been conclusive, with some reporting apparent sex differences across the life course [6] and birth cohorts [7, 8] and others reporting none [6, 8–12]. There is also a lack of published findings exploring ethnic differences in Asian populations. Therefore, this study explores the sex and ethnic variations in trajectories of body mass index and waist circumference among non-institutionalized Malaysian adults from 1996 to 2015 using a multilevel model. This model can fully utilize data from multiple waves of population-based cross-sectional studies to study changes in health status over time.

## Methods

### Study sample

A total of 94,537 nationally representative respondents aged 18–80 years were pooled from four serial population-based cross-sectional National Health and Morbidity Surveys (NHMS). These surveys were conducted in 1996 (*n* = 22,631), 2006 (*n* = 34,184), 2011 (*n* = 18,017), and 2015 (*n* = 19,705). The NHMS ’s samples were selected via a complex, two-stage stratified, probability-proportional-to-size sampling method [4]. The first-stage stratification was performed by states, and the second-stage was by urban/rural localities. These NHMSs covered both urban and rural areas across 13 states (Penang, Perlis, Kedah, Perak, Selangor, Negeri Sembilan, Melaka, Johor, Kelantan, Terengganu, Pahang, Sabah, Sarawak) and 2 Federal Territories (Kuala Lumpur and Putrajaya) in Malaysia. Data collection was conducted from March to July of each survey year. These series of NHMSs had high response rates ranging from 93.0% to 96.9%.

### BMI and waist circumference

Body weight was measured in light indoor clothing without shoes using a Tanita digital lithium weighing scale (Tanita 318, Japan in NHMS 1996 & 2006; Tanita HD319 in NHMS 2011 & 2015) to the nearest 0.1 kg. Standing height was measured without shoes using a SECA portable body meter (SECA 206, Germany in NHMS 1996, 2006 & 2011; SECA 213 in NHMS 2015) to the nearest 0.1 cm. Both weight and height measurements were measured twice in NHMS 2006, 2011, and 2015 and averaged values were used for analyses.

Data on waist circumference was only available in three of the NHMSs (2006, 2011, and 2015). Waist circumferences were taken midway between the inferior margin of the last rib and the crest of the ilium in a horizontal plane with the respondent’s feet 25 to 30 cm apart. Measurements were taken twice and averaged.

### Age, period, and cohort

The respondent’s age was derived by subtracting the date of birth recorded in the respondent’s nationality identity card from the interview date. Period refers to the NHMS survey year. The year of birth (birth cohort) was derived using the formula Cohort (years) = Period - Age.

### Statistical analysis

General and abdominal obesity were reported as weighted prevalence (%, 95% confidence interval) after adjusting for post-stratification weights. Body mass index (kg/m^2^) and waist circumference (cm) were summarized as mean (95% confidence interval). All analyses were stratified by survey years.

The extended hierarchical age-period-cohort (HAPC) analysis was used to model the BMI and waist circumference trajectories. This model operationalizes period and cohort as cross-classified contextual settings in which individual characteristics such as age reside [13]. The fixed part of the model incorporated the age and cohort effects based on the strong biological basis for an age effect and the progressively obesogenic environment over subsequent birth cohorts. The study period effect was excluded from the fixed part of the model to avoid mathematical dependency between APC. We assumed no major contemporaneous events occurred in the Malaysian population between 1996 and 2015. However, period effects were incorporated as random effects into the APC models to address secular changes (such as changes in health policies and health promotion programs) for valid inferences of age and cohort trajectories.

Model fit for the fixed part of the BMI and waist circumference models was assessed based on the significance of the likelihood ratio (LR) tests when covariates (age, cohort, ethnicity, polynomial terms for age and cohort, and the respective interaction terms between ethnicity and age, and, ethnicity and cohort) were added, one at a time, into the models. Models for waist circumference were further adjusted for weight and height.

All models were stratified by sex and fitted using the *mixed* program in STATA version 14 (StataCorp., College Station, TX, USA) and adjusted for post-stratification sampling weights. An additional geographical identifier, state-by-locality (i.e., state stratified by rural and urban locality), was added to all models, respectively, thereby extending the model to a four-level cross-classified model (Appendix I).

### Sensitivity analysis

Sensitivity analyses of BMI and waist circumference trajectories across ages were performed based on the alternative assumption of no linear cohort effects (age-period model). These analyses examined the possible presence of period effect such that the obesogenic environments could have affected the entire population at the times they existed, independent of their age and cohort.

## Results

The prevalence of both general and abdominal obesity has been increasing from 1996–2015 (Appendix II). Generally, Malaysian adults’ mean BMI (Appendix III) and waist circumference (Appendix V) have increased from 1996–2015 in both sexes and across ethnicities, age groups, cohort groups, and localities.

**Fig. 1:**
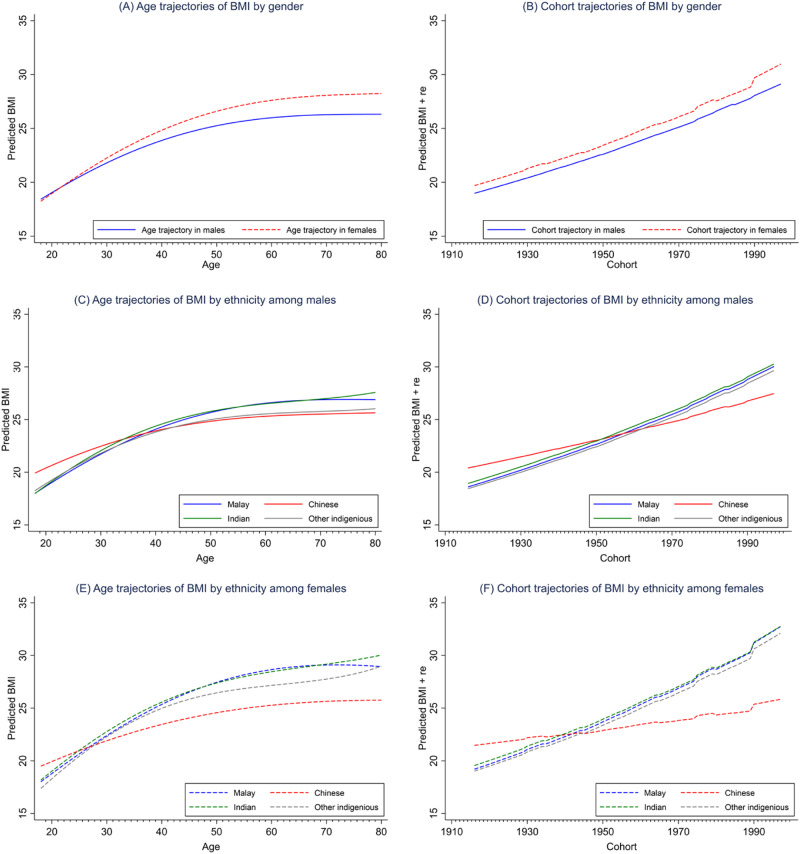
BMI trajectories across age and cohort, by sex and ethnicity. From left to right, top to bottom, the graph illustrates the BMI trajectories (**A**) across age by sex; (**B**) across year of birth (cohort) by sex; (**C**) across age by ethnicity in males; (**D**) across year of birth (cohort) by ethnicity in males; (**E**) across age by ethnicity in females; and (**F**) across year of birth (cohort) by ethnicity in females.

**Fig. 2:**
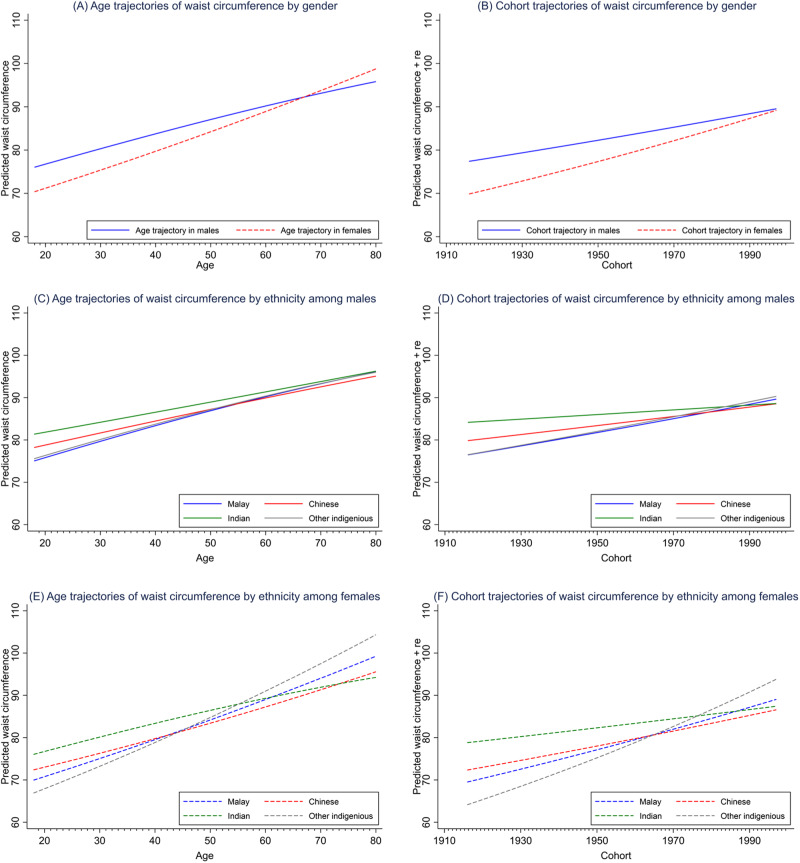
Waist circumference across age and cohort, by sex and ethnicity. From left to right, top to bottom, the graph illustrates the waist circumference trajectories (**A**) across age by sex; (**B**) across year of birth (cohort) by sex; (**C**) across age by ethnicity in males; (**D**) across year of birth (cohort) by ethnicity in males; (**E**) across age by ethnicity in females; and (**F**) across year of birth (cohort) by ethnicity in females.

Results from the sex-stratified APC analysis revealed that the BMI of Malaysian adults (both males and females) increased steeply from age 18 to 60 and then plateaued (Fig. 1A). Waist circumference, on the other hand, increased monotonically across the life course (Fig. 2A). Females have more profound BMI and waist circumference increases than their male counterparts across the life course (Figs. 1A and 2A) and birth cohorts (Figs. 1B and 2B).

The age and cohort trajectories of BMI and waist circumference also varied by ethnicity. The Chinese, compared to the other ethnic groups, had the least profound increasing trajectories of BMI and waist circumference across age (Fig. 1C,E for BMI; Fig. 2C,E for waist circumference) and birth cohorts (Fig. 1D,F for BMI; Fig. 2D,F for waist circumference) in both sexes. Such ethnic differences widened as people aged and cohort recency increased and were more pronounced among the females (Fig. 1E,F for BMI; Fig. 2E,F for waist circumference) than their male counterparts.

Sensitivity analysis revealed that alternative models assuming no cohort effect have poorer model fit than the primary models assuming no period effect (data not shown). We observed U-shaped age trajectories with sharp drops in BMI in both sexes at about age 50. For waist circumference, the estimated waist circumferences in males across the life course were below the cut-off point for abdominal obesity (Appendix XI).

## Discussion

In this study, the age trajectory of BMI increased steeply during adulthood and plateaued after age 60. The increasing trajectory in early adulthood was similar to previous APC studies on obesity [12] and mean BMI [9–11, 14–17]. However, their trajectories varied in the older age groups. A few studies reported a decreasing [8–12], whereas others reported a plateauing trend [14–16]. Our findings are consistent with the existing knowledge of decreased BMI among older people due to loss of skeletal muscle mass [18], reduced appetite, and malnourishment/poor nutrient intake [19]. In contrast, monotonic increasing age trajectories of waist circumference, which concurred with findings from a Norwegian study [20], were observed in both sexes. These increasing age trajectories can be attributed to the age-related increase in body fat, as studies have shown that the percentage of body fat increases with age until 80 years old [21] by about 1% per decade [22].

The monotonic increasing cohort trajectories of BMI and waist circumference observed in the present study were consistent with most studies reported elsewhere [6, 8, 9, 12, 14, 16, 23]. Cohort effects refer to inter-generational differences in experiences, exposures, behaviors, and socioeconomic factors between individuals of different birth cohorts. These differential exposures could have affected the habits and health behaviors in their formative years and persisted throughout their life course, thus leading to divergent health outcomes between different generations.

For instance, older cohorts born before the industrialization (the 1970s) and globalization era (1980s–1990s) may have experienced higher food scarcity, more labor-intensive occupations, and more physically active modes of transportation [24]. Therefore, they were less likely to be exposed to obesogenic risk factors such as uptake of energy-dense food, physical inactivity and sedentarism. In contrast, the progressively obesogenic environment may have predisposed the younger generations (particularly the Millennials or Generation Y born between 1981–1996) [8] to higher BMI and waist circumference than their counterparts in preceding cohorts. Possible reasons include increased availability, accessibility, and affordability of energy-dense nutrient-dilute foods, shift from labor- to capital-intensive occupations, increased screen time, and reduced active commute.

In the present study, a more profound increasing age trajectory of BMI [14] was observed among women, particularly as they age. This finding concurred with those reported in a cohort study among the rural population of The Netherlands. Evidence from a series of cross-sectional surveys in England demonstrated a more pronounced inverse association between BMI and height in older adults and women than in men, with little change over time [25]. Therefore, this increasing sexual dimorphism in the BMI–height associations could have led to the observed sex divergence of BMI trajectories over the life course, particularly during late adulthood.

On the other hand, other studies in high-income and upper-middle-income countries did not observe differences in age trajectories by sex for mean BMI [10, 11, 15] and prevalence of obesity [12]. These findings were in keeping with those observed from the Global Health Observatory, where equitable obesity rates between men and women were observed among most developed nations [26]. According to Grantham & Henneberg’s estrogen hypothesis, the preponderance of men’s exposure to environmental estrogen-like substances, such as xenoestrogen in soy products and polyvinyl chloride, that are related to the superfluous nature of developed nations, could have “feminized” the men, resulting in equitable obesity rates between men and women [27], particularly among those born in more recent cohorts.

We also observed sex variations in age and cohort trajectories of BMI and waist circumference: sex divergences in age and cohort trajectories of BMI but sex convergences in age and cohort trajectories of waist circumference. Sex dimorphism in fat distribution [28, 29] and physiological differences [30] may explain these variations. Since women tend to accrue more weight after menopause, they may be more likely to have higher BMI than men, thus explaining the increasing sex divergences in BMI trajectories as they age. On the contrary, men tend to store fat in visceral adipose tissue (VAT) in the deep abdominal region, compared to premenopausal women who preferentially keep excess fat in the subcutaneous adipose tissue (SAT) depots surrounding low extremities such as hips and thighs [31], thus predisposing men to greater waist circumference than premenopausal women. However, as age advances, SAT decreases, and VAT increases with age [22], with women having almost double increases in mean waist circumference than men [32]. Therefore, sex differences in VAT diminished as they age, resulting in the sex convergence in waist circumference over the life course.

The present study revealed that the age and cohort trajectories of BMI and waist circumference varied by ethnicity. Chinese had the least pronounced increasing trajectories of BMI. A recent APC study using four national longitudinal cohort studies also observed persistent ethnic differences in BMI trajectories across the life course between the Black, Hispanic and White [9]. Another APC study among the New Zealand population found ethnic differences in BMI trajectories between the Maori and non-Maori populations, with an increasing cohort trajectory only observed among the Maori [11]. For waist circumference, despite an almost similar magnitude of trajectories between Chinese and Indians, Indians had the highest overall waist circumference. This finding concurred with that reported in a local study where Indians have a greater likelihood of abdominal obesity than other ethnic groups across the life course [33].

Ethnic heterogeneities across the life course and birth cohorts can be attributable to the well-established ethnic variations in total body fat (TBF) percentage [34] and SAT and VAT fat depots [35]. Besides, the unique dietary habits among ethnic groups could also be the contributing factors. A greater preference for healthy-based over the Western-based (high in fat, sugar, and salts) food pattern among Chinese adolescents than their Malay counterparts [36] may likely explain healthier BMI and waist circumference trajectories among the Chinese, particularly those born more recently. In contrast, higher content of carbohydrates, saturated fatty acids, and trans fatty acids in the Indian diet may contribute to higher waist circumference across ages and cohorts [35].

Besides, genetic predisposition to certain diseases could also contribute to ethnic differences. Several ethnic-specific single nucleotide polymorphisms (SNPs) or single gene mutations associated with obesity have been identified [37]. In addition, racial/ethnic differentials in C-reactive protein (CRP) levels, a known risk factor of abdominal obesity [38], could be at play. Studies among the multi-ethnic US and Canadian populations had unequivocally reported that the Chinese had the lowest mean CRP level compared to other ethnicities such as Europeans, South Asians, aborigines [39], Caucasians, African Americans, and Hispanics [40]. These findings likely explain the more favorable obesity trajectories among the Chinese in the present study, who had the least profound increasing BMI and waist circumference trajectories, particularly among older adults and those born in more recent cohorts.

It is worth noting that due to the APC identification problem, strong assumptions must be made to discern the APC effect. These assumptions or constraints, however, cannot be made on the basis of the data [41]. To determine either cohort or period is more likely to be the driving temporal factor of obesity, one could compare the age trajectories produced and deduce which seems more plausible [41]. In our case, we would argue that the age trajectories of BMI and waist circumference predicted from the primary models that assumed no period effect are theoretically plausible compared to those predicted from the alternative models that assumed no cohort effect.

The age trajectories of BMI and waist circumference predicted from the primary models concurred with those reported in previous studies, where mean BMI [42] and waist circumference [43] increased with age until 60 to 70. Such trajectories are also consistent with ageing-related body composition changes such that body fat develops up to the eighth decade of life and reduces afterward [44].

On the other hand, the age trajectories of BMI, predicted from the alternative model, resembled the parabolic age trajectory of obesity among the U.S. adult population in a previous HAPC study that explicitly assumed no cohort effect [45]. As also argued by Bell and Jones [41], while BMI is known to be negatively associated with advancing age due to sarcopenia and survival bias; however, the relatively sharp and early decline in BMI and waist circumference at age 50, as observed in the alternative model (Appendix XI), are rather unlikely. Furthermore, the less profound age trajectories from the alternative models depict a much lower predicted mean BMI and waist circumference (in fact, well below the BMI cut-off point of 25.0 kg/m^2^ for overweight and waist circumference cut-off of 90 cm for men) among the Malaysian adult population, which is, again, unlikely given the fact that about 45% of Malaysia adults are overweight (Appendix II).

## Conclusion

The present findings add knowledge to the literature on obesity prevention and reducing ethnic disparities by identifying young adults of the most recent cohorts (the Millennials and Gen-Yers) as the high-risk sub-populations when BMI and waist circumference rapidly increased, and ethnic disparities emerged. The increasing BMI and waist circumference trajectories with cohort recency, regardless of age and ethnic groups, are alarming and deserve great attention as these trends are expected to increase in the foreseeable future.

The sex and ethnic divergence in BMI trajectories after middle adulthood suggests that the universal BMI cut-off of 25.0 kg/m^2^ for overweight and 30 kg/m^2^ for obesity for adults of all ages and sexes may not be appropriate, especially for females and older Malaysians. Therefore, future studies are needed to examine the sex-stratified association between BMI and waist circumference with morbidity and mortality among middle-aged and older adults and to propose sex- and, perhaps, ethnic-specific cut-offs for overweight and obesity for the Malaysian population. Such studies are also pertinent for a healthy ageing population. On the other hand, the less profound uptrends in BMI and waist circumference among the Chinese late Gen-Xers and Gen-Yers warrant further investigations to identify potential protecting factors for obesity prevention among the Malaysian population.

The main strength of the present study is the use of a pseudo-longitudinal research design by pooling a series of four nationally representative cross-sectional studies and a multilevel model to illuminate the obesity trajectories across a broad spectrum of developmental stages, encompassing late adolescence, young, middle and late adulthood and a wide span of birth cohorts. In this era of big data analytics, this study demonstrated the optimum use of readily available nationally representative cross-sectional data in generating results comparable to those in traditional longitudinal data [46]. This approach is particularly useful in resource-limited countries. Secondly, weight, height, and waist circumference were measured by trained nurses, thus minimizing self-report and measurement bias. Thirdly, APC analysis was performed to account for the confounding effects of age, period, and cohort for valid inferences of age and cohort trajectories.

Nonetheless, there are also a few limitations. First, the APC analysis is descriptive; therefore, the underlying factors causing the increasing BMI and waist circumference trajectories across the life course and cohorts remained a topic for future research. Second, due to the inherent multicollinearity between APC, we explicitly assumed no period effect (instead of age or cohort effect). However, we contend that this assumption, in the context of the obesity epidemic in Malaysia, is physiologically plausible compared to those predicted from the alternative models that assumed no cohort effect. Nevertheless, cautious attention is warranted for researchers that wish to adopt this model since the assumption made hereto (no period effect) is not a one-size-fits-all approach and other factors, such as the data structure, research aims, and contextual setting that are unique to each population, must be taken into account for HAPC model specification. Third, despite the flexibility of using a series of nationally representative, cross-sectional studies in APC studies to model health trajectories, it must be noted that these trajectories might differ from those generated from a proper longitudinal study where individuals are followed up for a period of time.

### Supplementary information

Supplementary figure and table legends

Appendix I

Appendix II

Appendix III

Appendix IV

Appendix V

Appendix VI

Appendix VII

Appendix VIII

Appendix IX

Appendix X

Appendix XI

Appendix XII

Appendix XIII

## Data Availability

Data sources and coding were deposited in the National Institutes of Health – Data Repository System (NIH-DaRS) at https://nihdars.nih.gov.my/. However, this information is only available upon request and is subject to approval by the Director General of Health Malaysia.
