# Correction: Body mass index and waist circumference trajectories across the life course and birth cohorts, 1996–2015 Malaysia: sex and ethnicity matter

**DOI:** 10.1038/s41366-023-01447-6

**Published:** 2023-12-22

**Authors:** Chien Huey Teh, Sanjay Rampal, Chee Cheong Kee, Omar Azahadi, Aris Tahir

**Affiliations:** 1 Centre for Epidemiology and Evidence-based Practice, Department of Social and Preventive Medicine, Faculty of Medicine, University of Malaya, 50603 Kuala Lumpur, Malaysia; 2 Institute for Medical Research, National Institutes of Health, Ministry of Health Malaysia, 40170 Setia Alam, Malaysia; 3 Sector for Biostatistics and Data Repository, National Institutes of Health, Ministry of Health Malaysia, 40170 Setia Alam, Malaysia

**Keywords:** Risk factors, Obesity, Epidemiology

Correction to: *International Journal of Obesity* 10.1038/s41366-023-01391-5, published online 13 October 2023

In the original article, the author’s name Chee Cheong Kee was incorrectly given as ‘Chee Keong Kee’. The original article has been corrected.

