# Supplementary material for: Body mass index and waist circumference trajectories across the life course and birth cohorts, 1996–2015 Malaysia: sex and ethnicity matter
Source: Int J Obes (Lond). 2023 Oct 13;47(12):1302–8. doi: 10.1038/s41366-023-01391-5 (PMC10663154; doi:10.1038/s41366-023-01391-5)
Supplement: Supplementary file 1 — Supplementary figure and table legends [file 41366_2023_1391_MOESM1_ESM.docx]

**Supplementary figure and table legends**

**Appendix I Model specifications for BMI and waist circumference**

# Appendix II Prevalence of general and abdominal obesity and distributions of BMI and waist circumference among Malaysian adults aged 18-80 years, NHMS 1996, 2006, 2011 and 2015

# Appendix III Mean BMI (kg/m^2^) of Malaysian adults by socio-demographics, NHMS 1996, 2006, 2011 and 2015

# Appendix IV Median and interquartile range (IQR) of BMI (kg/m^2^) among Malaysian adults, NHMS 1996, 2006, 2011 and 2015

# Appendix V Mean waist circumference (cm) of Malaysian adults by socio-demographics, NHMS 2006, 2011 and 2015

# Appendix VI Median and interquartile range (IQR) of waist circumference (cm) among Malaysian adults, NHMS 2006, 2011 and 2015

# Appendix VII Parameter estimates of the extended HAPC model of BMI among male adults aged 18-80 years in Malaysia (NHMS 1996, 2006, 2011 and 2015)

# Appendix VIII Parameter estimates of the extended HAPC model of BMI among female adults aged 18-80 years in Malaysia (NHMS 1996, 2006, 2011 and 2015)

# Appendix IX Parameter estimates of the extended HAPC model of waist circumference among male adults aged 18-80 years in Malaysia (NHMS 2006, 2011 and 2015)

# Appendix X Parameter estimates of the extended HAPC model of waist circumference among female adults aged 18-80 years in Malaysia (NHMS 2006, 2011 and 2015)

Appendix XI Sensitivity analysis (age-period model): BMI and waist circumference trajectories across age, by sex

Appendix XII BMI and waist circumference trajectories across age among Malays, by sex From left to right, top to bottom, the graph illustrates the BMI trajectories (A) across age by sex; and (B) across year of birth by sex; and waist circumference trajectories (C) across age by sex; and (D) across year of birth by sex

Appendix XIII BMI and waist circumference trajectories across age among Chinese, by sex From left to right, top to bottom, the graph illustrates the BMI trajectories (A) across age by sex; and (B) across year of birth by sex; and waist circumference trajectories (C) across age by sex; and (D) across year of birth by sex
