# Supplementary material for: Body mass index and waist circumference trajectories across the life course and birth cohorts, 1996–2015 Malaysia: sex and ethnicity matter
Source: Int J Obes (Lond). 2023 Oct 13;47(12):1302–8. doi: 10.1038/s41366-023-01391-5 (PMC10663154; doi:10.1038/s41366-023-01391-5)
Supplement: Supplementary file 2 — Appendix I [file 41366_2023_1391_MOESM2_ESM.docx]

$${lnBMI}_{i\left( jkm \right)}= \beta_{o}+\beta_{1}{Age}_{i\left( jkm \right)}+\beta_{2}{Age}_{i\left( jkm \right)}^{2}+ \beta_{3}{Age}_{i\left( jkm \right)}^{3}+ \beta_{4}{Cohort}_{i\left( jkm \right)} + \beta_{5}{Ethnicity}_{i\left( jkm \right)}+ \beta_{6}\left( Ethnicity x Age \right)_{i\left( jkm \right)}+ \beta_{7}\left( Ethnicity x {Age}^{2} \right)_{i\left( jkm \right)}+ \beta_{8}\left( Ethnicity x {Age}^{3} \right)_{i\left( jkm \right)}+ \beta_{9}\left( Ethnicity x Cohort \right)_{i\left( jkm \right)}+ \beta_{10}{Urban}_{i\left( jkm \right)}+ \beta_{11}{(Ethnicity x Urban)}_{i\left( jkm \right)}+ \mu_{1j}+\mu_{2k}+ \mu_{3m}+e_{i(jkm)}$$

$${lnWC}_{i\left( jkm \right)}= \beta_{o}+\beta_{1}{Age}_{i\left( jkm \right)}+\beta_{2}{Age}_{i\left( jkm \right)}^{2}+ \beta_{3}{Cohort}_{i\left( jkm \right)}+ \beta_{4}{Ethnicity}_{i\left( jkm \right)}++ \beta_{5}\left( Ethnicity x Age \right)_{i\left( jkm \right)}+ \beta_{6}\left( Ethnicity x {Age}^{2} \right)_{i\left( jkm \right)}+ \beta_{7}\left( Ethnicity x Cohort \right)_{i\left( jkm \right)}+ \beta_{8}{Urban}_{i\left( jkm \right)}+ \beta_{9}{(Ethnicity x Urban)}_{i\left( jkm \right)}+ \beta_{10}{Weight}_{i\left( jkm \right)}+ \beta_{11}{Height}_{i\left( jkm \right)}+ \mu_{1j}+\mu_{2k}+ \mu_{3m}+e_{i(jkm)}$$

, where *In*${BMI}_{i\left( jkm \right)}$ is the natural log-transformed values of BMI (*In*${WC}_{i\left( jkm \right)}$for waist circumference) of individual *i* that was born in cohort *j,* being surveyed in period *k*, and resided in state-by-locality *m*. The fixed coefficients $\beta_{1}$ to $\beta_{11}$ and a non-varying constant, $\beta_{o}$ _,_ constituted the fixed part of the model. The random part of the model is composed of $\mu_{1j}$, the random variation of the 5-year-interval cohort group *j*, $\mu_{2k}$, the random variation of period *k,* $\mu_{3m}$, the random variation of state-by-locality *m,* and $e_{i(jkm)}$, the residual error term due to variations not explained by the model. We assumed the residual error, random variations of period, cohort and state-by-locality follow normal distributions with mean = 0 and an estimated variance ($e_{i(jk)}, \sigma_{u2}^{2}, \sigma_{u2}^{2}, \sigma_{u3}^{2}$, each presented as random effect estimates at Appendix VII to X, respectively), as depicted below:

$$e_{i(jk)} \sim N \left( 0,\sigma_{e}^{2} \right), \mu_{1j} \sim N \left( 0,\sigma_{u1}^{2} \right), \mu_{2k} \sim N \left( 0,\sigma_{u2}^{2} \right), \mu_{3m} \sim N \left( 0,\sigma_{u3}^{2} \right)$$

Age and birth cohort were mean-centred at 40 and 1960, respectively, to reduce correlations between the linear and polynomial terms. Cohort were categorised into 5-year-interval in the random part of the model to adjust for autocorrelation between year of birth.
