# Supplementary material for: Body mass index and waist circumference trajectories across the life course and birth cohorts, 1996–2015 Malaysia: sex and ethnicity matter
Source: Int J Obes (Lond). 2023 Oct 13;47(12):1302–8. doi: 10.1038/s41366-023-01391-5 (PMC10663154; doi:10.1038/s41366-023-01391-5)
Supplement: Supplementary file 3 — Appendix II [file 41366_2023_1391_MOESM3_ESM.docx]

|  | **NHMS** | | | | | | | | | | |
| --- | --- | --- | --- | --- | --- | --- | --- | --- | --- | --- | --- |
|  | **1996** | | **2006** | | **2011** | | **2015** | | **Total** | | **N** |
|  | %^a^ | 95% CI | %^a^ | 95% CI | %^a^ | 95% CI | %^a^ | 95% CI | %^a^ | 95% CI |  |
|  | | | | | | | | | | | |
| **General obesity** BMI ≥ 25.0kg/m^2^ (WHO 1999) | 34.1 | [33.2-35.0] | 44.4 | [43.7-45.1] | 45.4 | [44.1-46.7] | 48.6 | [47.4-49.8] | 45.0 | [44.4-45.6] | 38 368 |
|  | | | | | | | | | | | |
| **General obesity** BMI ≥ 23.0kg/m^2^ (WHO 2004 Asian BMI) | 53.4 | [52.5-54.3] | 61.4 | [60.7-62.1] | 61.6 | [60.3-62.9] | 65.2 | [64.0-66.4] | 61.9 | [61.3-62.5] | 53 221 |
|  |  |  |  |  |  |  |  |  |  |  |  |
| **Abdominal obesity** ≥ ≥94cm in men; ≥80cm in women (WHO 2000) | *Data not collected* | | 36.6 | [35.9-37.3] | 39.4 | [38.0-40.7] | 42.4 | [41.1-43.6] | 39.8 | [39.1-40.5] | 27 377 |
|  |  |  |  |  |  |  |  |  |  |  |  |
| **Abdominal obesity** ≥90cm in men; ≥80cm in women (IDF 2006 Definition for Asian) | *Data not collected* | | 41.6 | [40.9-42.3] | 45.5 | [44.1-46.8] | 48.7 | [47.4-50.0] | 45.7 | [45.0-46.5] | 30 983 |
|  |  |  |  |  |  |  |  |  |  |  |  |

% - prevalence was weighted to be nationally representative

Data missingness based on BMI by NHMS: 9.4%; 4.4%; 7.1%; 6.9%

Data missingness based on waist circumference by NHMS: not applicable; 4.6%; 9.7; 7.0%
