# Supplementary material for: Body mass index and waist circumference trajectories across the life course and birth cohorts, 1996–2015 Malaysia: sex and ethnicity matter
Source: Int J Obes (Lond). 2023 Oct 13;47(12):1302–8. doi: 10.1038/s41366-023-01391-5 (PMC10663154; doi:10.1038/s41366-023-01391-5)
Supplement: Supplementary file 4 — Appendix III [file 41366_2023_1391_MOESM4_ESM.docx]

|  | **NHMS** | | | | | | | | | | |  |
| --- | --- | --- | --- | --- | --- | --- | --- | --- | --- | --- | --- | --- |
|  | **1996** | | **2006** | | **2011** | | **2015** | | **Total** | | **N** | |
|  | **Mean** | **95%CI** | **Mean** | **95%CI** | **Mean** | **95%CI** | **Mean** | **95%CI** | **Mean** | **95%CI** |  | |
| Overall | 23.8 | [23.7-23.9] | 24.9 | [24.9-25.0] | 25.1 | [25.0-25.3] | 25.6 | [25.4-25.7] | 25.1 | [25.0-25.2] | 86 500 | |
|  |  |  |  |  |  |  |  |  |  |  |  | |
| Sex |  |  |  |  |  |  |  |  |  |  |  | |
| Male | 23.3 | [23.3-23.4] | 24.3 | [24.2-24.4] | 24.8 | [24.6-25.0] | 25.2 | [25.0-25.3] | 24.7 | [24.6-24.8] | 41 349 | |
| Female | 24.2 | [24.1-24.3] | 25.5 | [25.4-25.6] | 25.5 | [25.3-25.6] | 26.0 | [25.9-26.2] | 25.5 | [25.4-25.6] | 44 894 | |
|  |  |  |  |  |  |  |  |  |  |  |  | |
| Ethnicity |  |  |  |  |  |  |  |  |  |  |  | |
| Malay | 24.0 | [23.8-24.1] | 25.4 | [25.3-25.5] | 25.8 | [25.6-26.0] | 26.1 | [26.0-26.3] | 25.6 | [25.5-25.7] | 46 380 | |
| Chinese | 23.5 | [23.4-23.7] | 24.1 | [24.0-24.3] | 24.1 | [23.9-24.3] | 24.5 | [24.3-24.8] | 24.2 | [24.1-24.3] | 18 172 | |
| Indian | 24.5 | [24.2-24.7] | 25.9 | [25.6-26.1] | 26.2 | [25.6-26.8] | 27.1 | [26.7-27.6] | 26.2 | [25.9-26.4] | 6 539 | |
| Other Bumiputra | 23.3 | [23.1-23.5] | 24.5 | [24.3-24.7] | 24.9 | [24.4-25.4] | 25.8 | [25.4-26.1] | 25.0 | [24.8-25.2] | 9 524 | |
| Others | 22.9 | [22.5-23.2] | 23.6 | [23.3-23.8] | 23.1 | [22.7-23.6] | 24.0 | [23.6-24.3] | 23.6 | [23.4-23.8] | 5 608 | |
|  |  |  |  |  |  |  |  |  |  |  |  | |
| Age (10-year interval) | | | | | | | | | | | |  |
| 18-29 | 23.3 | [21.5-25.0] | 23.2 | [23.0-23.3] | 23.8 | [23.5-24.0] | 24.3 | [24.1-24.5] | 23.8 | [23.7-24.0] | 17 352 | |
| 30-39 | 23.6 | [23.5-23.7] | 25.2 | [25.0-25.3] | 25.5 | [25.2-25.7] | 26.0 | [25.7-26.2] | 25.2 | [25.1-25.4] | 21 780 | |
| 40-49 | 24.3 | [24.2-24.4] | 26.0 | [25.9-26.1] | 26.2 | [25.9-26.5] | 26.4 | [26.2-26.6] | 25.9 | [25.8-26.0] | 20 058 | |
| 50-59 | 23.9 | [23.7-24.1] | 26.2 | [26.0-26.3] | 26.2 | [26.0-26.4] | 26.6 | [26.4-26.9] | 26.0 | [25.9-26.1] | 15 248 | |
| 60-69 | 23.4 | [23.2-23.6] | 25.1 | [25.0-25.3] | 25.7 | [25.4-26.0] | 26.2 | [26.0-26.5] | 25.4 | [25.3-25.6] | 8 557 | |
| 70-79 | 22.4 | [22.1-22.7] | 24.0 | [23.8-24.3] | 24.4 | [24.0-24.8] | 24.9 | [24.5-25.3] | 24.3 | [24.1-24.5] | 3 370 | |
| 80 & above | 21.5 | [20.4-22.6] | 22.4 | [21.4-23.4] | 24.1 | [22.3-25.8] | 23.2 | [21.5-24.8] | 23.0 | [22.1-23.9] | 135 | |
|  |  |  |  |  |  |  |  |  |  |  |  | |
| Year of birth (10-year interval) | | | | | | | | | | | |  |
| 1929 and earlier | 22.6 | [22.4-22.9] | 23.0 | [22.5-23.5] | 0.0 | - | 0.0 | - | 22.7 | [22.5-22.9] | 1 447 | |
| 1930-1939 | 23.5 | [23.3-23.7] | 24.5 | [24.3-24.8] | 24.4 | [24.0-24.9] | 24.2 | [23.6-24.8] | 24.1 | [23.9-24.2] | 4 961 | |
| 1940-1949 | 24.1 | [24.0-24.3] | 25.5 | [25.4-25.7] | 25.5 | [25.1-25.8] | 25.6 | [25.2-25.9] | 25.1 | [25.0-25.2] | 10 288 | |
| 1950-1959 | 24.2 | [24.1-24.3] | 26.3 | [26.2-26.4] | 26.2 | [25.9-26.5] | 26.5 | [26.3-26.8] | 25.8 | [25.7-25.9] | 18 060 | |
| 1960-1969 | 23.5 | [23.4-23.6] | 25.8 | [25.7-25.9] | 26.2 | [25.9-26.5] | 26.6 | [26.3-26.8] | 25.8 | [25.6-25.9] | 20 020 | |
| 1970-1979 | 24.5 | [24.5-24.5] | 24.8 | [24.6-24.9] | 25.7 | [25.5-25.9] | 26.4 | [26.1-26.6] | 25.7 | [25.6-25.8] | 13 474 | |
| 1980-1989 | 0.0 | - | 22.8 | [22.7-22.9] | 24.4 | [24.1-24.6] | 25.5 | [25.2-25.7] | 24.5 | [24.3-24.6] | 13 983 | |
| 1990-1997 | 0.0 | - | 0.0 | - | 22.8 | [22.5-23.2] | 23.8 | [23.6-24.1] | 23.5 | [23.3-23.7] | 4 267 | |
|  |  |  |  |  |  |  |  |  |  |  |  | |
| Locality |  |  |  |  |  |  |  |  |  |  |  | |
| Rural | 23.6 | [23.4-23.7] | 24.8 | [24.7-24.9] | 25.0 | [24.8-25.3] | 25.4 | [25.2-25.7] | 24.8 | [24.7-24.9] | 36 304 | |
| Urban | 24.0 | [23.9-24.1] | 25.0 | [24.9-25.1] | 25.1 | [25.0-25.3] | 25.6 | [25.5-25.8] | 25.2 | [25.1-25.3] | 50 196 | |
|  |  |  |  |  |  |  |  |  |  |  |  | |
|  |  |  |  |  |  |  |  |  |  |  |  | |
