# Supplementary material for: Body mass index and waist circumference trajectories across the life course and birth cohorts, 1996–2015 Malaysia: sex and ethnicity matter
Source: Int J Obes (Lond). 2023 Oct 13;47(12):1302–8. doi: 10.1038/s41366-023-01391-5 (PMC10663154; doi:10.1038/s41366-023-01391-5)
Supplement: Supplementary file 5 — Appendix IV [file 41366_2023_1391_MOESM5_ESM.docx]

|  | **NHMS** | | | | | | | | | | |  |
| --- | --- | --- | --- | --- | --- | --- | --- | --- | --- | --- | --- | --- |
|  | **1996** | | **2006** | | **2011** | | **2015** | | **Total** | | **N** | |
|  | **Median** | **IQR** | **Median** | **IQR** | **Median** | **IQR** | **Median** | **IQR** | **Median** | **IQR** |  | |
| Overall | 23.3 | 5.3 | 24.3 | 6.5 | 24.8 | 6.6 | 25.4 | 6.9 | 24.3 | 6.4 | 86 500 | |
|  |  |  |  |  |  |  |  |  |  |  |  | |
| Sex |  |  |  |  |  |  |  |  |  |  |  | |
| Male | 23.0 | 4.9 | 23.8 | 5.9 | 24.4 | 6.2 | 24.9 | 6.4 | 23.9 | 5.9 | 41 349 | |
| Female | 23.6 | 5.7 | 24.8 | 7.0 | 25.2 | 7.1 | 25.9 | 7.3 | 24.8 | 6.9 | 44 894 | |
|  |  |  |  |  |  |  |  |  |  |  |  | |
| Ethnicity |  |  |  |  |  |  |  |  |  |  |  | |
| Malay | 23.5 | 5.5 | 24.8 | 6.9 | 25.3 | 7.0 | 25.8 | 7.1 | 24.8 | 6.8 | 46 380 | |
| Chinese | 23.2 | 4.8 | 23.7 | 5.5 | 23.9 | 5.7 | 24.3 | 5.8 | 23.6 | 5.4 | 18 172 | |
| Indian | 23.9 | 5.3 | 25.3 | 6.6 | 25.6 | 7.0 | 26.5 | 7.0 | 25.2 | 6.6 | 6 539 | |
| Other Bumiputra | 22.9 | 5.2 | 23.8 | 6.0 | 24.5 | 6.1 | 25.3 | 6.5 | 23.9 | 6.0 | 9 524 | |
| Others | 22.4 | 4.7 | 22.7 | 5.4 | 23.0 | 5.4 | 23.7 | 5.6 | 22.9 | 5.3 | 5 608 | |
|  |  |  |  |  |  |  |  |  |  |  |  | |
| Age (10-year interval) | | | | | | | | | | | |  |
| 18-29 | 23 | 4.7 | 22 | 5.8 | 22.6 | 6.9 | 23.2 | 7.3 | 22.4 | 6.5 | 17 352 | |
| 30-39 | 23.1 | 5.2 | 24.4 | 6.3 | 25.1 | 6.7 | 25.6 | 7.0 | 24.1 | 6.2 | 21 780 | |
| 40-49 | 23.9 | 5.3 | 25.5 | 6.0 | 25.8 | 6.0 | 26.4 | 6.2 | 25.1 | 5.9 | 20 058 | |
| 50-59 | 23.4 | 5.3 | 25.7 | 5.8 | 26 | 6.1 | 26.3 | 6.1 | 25.3 | 6.1 | 15 248 | |
| 60-69 | 22.8 | 5.2 | 24.8 | 5.8 | 25 | 5.9 | 25.8 | 5.9 | 24.6 | 5.9 | 8 557 | |
| 70-79 | 22 | 5.0 | 23.6 | 5.5 | 24.1 | 5.3 | 24.5 | 5.8 | 23.5 | 5.6 | 3 370 | |
| 80 & above | 20.6 | 4.6 | 22.2 | 4.1 | 23.3 | 6.0 | 23.4 | 5.3 | 22.3 | 5.5 | 135 | |
|  |  |  |  |  |  |  |  |  |  |  |  | |
| Year of birth (10-year interval) | | | | | | | | | | | |  |
| 1929 and earlier | 22.2 | 5.0 | 22.4 | 5.0 | 0.0 | - | 0.0 | - | 22.2 | 5.1 | 1 447 | |
| 1930-1939 | 23.0 | 5.0 | 24.0 | 5.9 | 23.7 | 5.2 | 23.8 | 5.1 | 23.5 | 5.4 | 4 961 | |
| 1940-1949 | 23.6 | 5.3 | 25.0 | 5.8 | 25.0 | 5.7 | 25.1 | 6.0 | 24.5 | 5.7 | 10 288 | |
| 1950-1959 | 23.7 | 5.3 | 25.8 | 5.8 | 25.8 | 6.3 | 26.0 | 6.0 | 25.1 | 5.9 | 18 060 | |
| 1960-1969 | 22.9 | 5.1 | 25.2 | 6.1 | 25.9 | 6.0 | 26.3 | 6.1 | 24.8 | 6.1 | 20 020 | |
| 1970-1979 | 24.5 | 0.0 | 23.9 | 6.4 | 25.3 | 6.5 | 26.3 | 6.5 | 24.9 | 6.7 | 13 474 | |
| 1980-1989 | 0.0 | - | 21.6 | 5.5 | 23.3 | 7.0 | 24.9 | 7.3 | 22.8 | 6.8 | 13 983 | |
| 1990-1997 | 0.0 | - | 0.0 | - | 21.5 | 6.3 | 22.6 | 6.9 | 22.3 | 6.8 | 4 267 | |
|  |  |  |  |  |  |  |  |  |  |  |  | |
| Locality |  |  |  |  |  |  |  |  |  |  |  | |
| Rural | 23.0 | 5.3 | 24.1 | 6.6 | 24.8 | 6.6 | 25.4 | 7.1 | 24.2 | 6.5 | 36 304 | |
| Urban | 23.5 | 5.2 | 24.4 | 6.3 | 24.7 | 6.6 | 25.4 | 6.6 | 24.4 | 6.2 | 50 196 | |
|  |  |  |  |  |  |  |  |  |  |  |  | |
|  |  |  |  |  |  |  |  |  |  |  |  | |
