# Supplementary material for: Body mass index and waist circumference trajectories across the life course and birth cohorts, 1996–2015 Malaysia: sex and ethnicity matter
Source: Int J Obes (Lond). 2023 Oct 13;47(12):1302–8. doi: 10.1038/s41366-023-01391-5 (PMC10663154; doi:10.1038/s41366-023-01391-5)
Supplement: Supplementary file 6 — Appendix V [file 41366_2023_1391_MOESM6_ESM.docx]

|  | **NHMS** | | | | | | | | |
| --- | --- | --- | --- | --- | --- | --- | --- | --- | --- |
|  | **2006** | | **2011** | | **2015** | | **Total** | | **N** |
|  | **Mean** | **95%CI** | **Mean** | **95%CI** | **Mean** | **95%CI** | **Mean** | **95%CI** |  |
| Overall | 82.1 | [81.9-82.3] | 84.0 | [83.6-84.4] | 85.3 | [84.9-85.6] | 84.0 | [83.8-84.2] | 66 777 |
|  |  |  |  |  |  |  |  |  |  |
| Sex |  |  |  |  |  |  |  |  |  |
| Male | 84.1 | [83.8-84.4] | 86.0 | [85.5-86.5] | 86.8 | [86.3-87.3] | 85.9 | [85.6-86.2] | 31 360 |
| Female | 80.4 | [80.2-80.6] | 81.9 | [81.4-82.4] | 83.6 | [83.1-84.0] | 82.1 | [81.8-82.3] | 35 417 |
|  |  |  |  |  |  |  |  |  |  |
| Ethnicity |  |  |  |  |  |  |  |  |  |
| Malay | 82.4 | [82.1-82.7] | 84.9 | [84.4-85.4] | 85.7 | [85.2-86.1] | 84.5 | [84.3-84.8] | 37 473 |
| Chinese | 82.2 | [81.8-82.6] | 83.6 | [82.9-84.2] | 84.3 | [83.6-85.1] | 83.5 | [83.1-83.9] | 12 443 |
| Indian | 86.6 | [85.9-87.2] | 89.5 | [88.3-90.6] | 90.3 | [89.1-91.6] | 88.9 | [88.3-89.6] | 5 194 |
| Other Bumiputra | 79.8 | [79.3-80.3] | 81.8 | [80.4-83.1] | 85.2 | [84.2-86.3] | 82.7 | [82.0-83.4] | 6 950 |
| Others | 77.5 | [76.9-78.2] | 77.6 | [76.2-78.9] | 82.2 | [81.2-83.2] | 79.8 | [79.1-80.5] | 4 717 |
|  |  |  |  |  |  |  |  |  |  |
| Age (10-year interval) |  |  |  |  |  |  |  |  |  |
| 18-29 | 76.0 | [75.7-76.3] | 79.2 | [78.5-79.8] | 80.7 | [80.1-81.2] | 79.1 | [78.7-79.4] | 17 591 |
| 30-39 | 81.8 | [81.5-82.2] | 84.8 | [84.2-85.5] | 85.7 | [85.1-86.3] | 84.5 | [84.1-84.8] | 13 645 |
| 40-49 | 84.7 | [84.4-85.1] | 86.6 | [86.0-87.2] | 87.1 | [86.5-87.6] | 86.2 | [85.9-86.5] | 14 029 |
| 50-59 | 86.7 | [86.3-87.0] | 88.6 | [88.0-89.2] | 89.7 | [89.0-90.3] | 88.4 | [88.1-88.7] | 11 756 |
| 60-69 | 85.3 | [84.8-85.7] | 88.5 | [87.7-89.4] | 89.7 | [88.9-90.4] | 88.0 | [87.6-88.5] | 6 672 |
| 70-79 | 82.8 | [82.0-83.6] | 86.1 | [85.0-87.2] | 88.0 | [86.9-89.1] | 85.9 | [85.3-86.5] | 2 968 |
| 80 & above | 79.4 | [76.5-82.4] | 87.0 | [81.5-92.5] | 83.8 | [79.6-88.1] | 83.7 | [80.9-86.5] | 116 |
|  |  |  |  |  |  |  |  |  |  |
| Year of birth (10-year interval) | | | | | | | | | |
| 1929 and earlier | 81.2 | [79.6-82.8] | 0.0 | - | 0.0 | - | 81.2 | [79.6-82.8] | 279 |
| 1930-1939 | 83.7 | [83.1-84.3] | 87.0 | [85.7-88.2] | 87.0 | [85.6-88.4] | 85.3 | [84.7-85.9] | 2 707 |
| 1940-1949 | 86.1 | [85.6-86.5] | 87.7 | [86.8-88.6] | 88.7 | [87.8-89.6] | 87.3 | [86.9-87.7] | 6 307 |
| 1950-1959 | 86.3 | [86.0-86.7] | 88.8 | [88.2-89.5] | 90.1 | [89.5-90.8] | 88.3 | [88.0-88.6] | 11 408 |
| 1960-1969 | 84.0 | [83.7-84.3] | 87.1 | [86.4-87.7] | 88.5 | [87.9-89.0] | 86.6 | [86.2-86.9] | 14 150 |
| 1970-1979 | 80.7 | [80.4-81.0] | 85.2 | [84.6-85.9] | 86.9 | [86.2-87.5] | 84.6 | [84.3-85.0] | 13 439 |
| 1980-1989 | 75.0 | [74.6-75.4] | 81.0 | [80.4-81.6] | 84.4 | [83.9-85.0] | 81.1 | [80.7-81.5] | 14 171 |
| 1990-1997 | 0.0 | - | 76.3 | [75.3-77.3] | 79.2 | [78.6-79.9] | 78.4 | [77.8-78.9] | 4 316 |
|  |  |  |  |  |  |  |  |  |  |
| Locality |  |  |  |  |  |  |  |  |  |
| Rural | 81.1 | [80.8-81.4] | 82.9 | [82.2-83.6] | 84.2 | [83.6-84.9] | 82.8 | [82.4-83.1] | 27 636 |
| Urban | 82.6 | [82.3-82.9] | 84.4 | [83.9-84.9] | 85.6 | [85.2-86.0] | 84.5 | [84.2-84.7] | 39 141 |
|  |  |  |  |  |  |  |  |  |  |
|  |  |  |  |  |  |  |  |  |  |
