# Supplementary material for: Body mass index and waist circumference trajectories across the life course and birth cohorts, 1996–2015 Malaysia: sex and ethnicity matter
Source: Int J Obes (Lond). 2023 Oct 13;47(12):1302–8. doi: 10.1038/s41366-023-01391-5 (PMC10663154; doi:10.1038/s41366-023-01391-5)
Supplement: Supplementary file 7 — Appendix VI [file 41366_2023_1391_MOESM7_ESM.docx]

|  |  |  |  |  |  |  |  |  |  |
| --- | --- | --- | --- | --- | --- | --- | --- | --- | --- |
|  | **NHMS** | | | | | |  | |  |
|  | **2006** | | **2011** | | **2015** | | **Total** | | **N** |
|  | **Median** | **IQR** | **Median** | **IQR** | **Median** | **IQR** | **Median** | **IQR** |  |
| Overall | 24.3 | 6.5 | 24.8 | 6.6 | 25.4 | 6.9 | 24.3 | 6.4 | 66 777 |
|  |  |  |  |  |  |  |  |  |  |
| Sex |  |  |  |  |  |  |  |  |  |
| Male | 83.4 | 17.6 | 86.0 | 17.0 | 87.0 | 16.0 | 85.0 | 17.1 | 31 360 |
| Female | 79.5 | 18.0 | 82.0 | 18.5 | 85.0 | 17.1 | 81.9 | 18.0 | 35 417 |
|  |  |  |  |  |  |  |  |  |  |
| Ethnicity |  |  |  |  |  |  |  |  |  |
| Malay | 82.0 | 18.0 | 85.0 | 18.5 | 86.0 | 17.2 | 84.0 | 18.0 | 37 473 |
| Chinese | 82.0 | 16.0 | 83.5 | 17.0 | 85.0 | 15.1 | 83.0 | 16.0 | 12 443 |
| Indian | 87.0 | 17.9 | 90.0 | 18.0 | 89.3 | 16.5 | 88.0 | 17.0 | 5 194 |
| Other Bumiputra | 78.7 | 16.6 | 81.0 | 17.0 | 85.0 | 15.9 | 81.0 | 16.9 | 6 950 |
| Others | 76.0 | 15.0 | 78.0 | 15.5 | 82.0 | 14.5 | 78.0 | 15.0 | 4 717 |
|  |  |  |  |  |  |  |  |  |  |
| Age (10-year interval) | | | | | | | | | |
| 18-29 | 73.8 | 15.6 | 77.0 | 17.1 | 79.0 | 17.1 | 76.0 | 16.8 | 17 591 |
| 30-39 | 81.0 | 16.1 | 84.0 | 16.7 | 85.0 | 16.0 | 83.0 | 16.0 | 13 645 |
| 40-49 | 84.1 | 15.7 | 86.0 | 15.3 | 87.0 | 14.0 | 85.5 | 15.0 | 14 029 |
| 50-59 | 87.0 | 15.1 | 88.4 | 16.1 | 89.3 | 15.0 | 88.0 | 15.9 | 11 756 |
| 60-69 | 85.5 | 17.2 | 87.3 | 16.0 | 89.5 | 15.0 | 87.0 | 16.0 | 6 672 |
| 70-79 | 83.0 | 17.0 | 86.0 | 15.7 | 87.0 | 16.0 | 85.0 | 16.9 | 2 968 |
| 80 & above | 78.0 | 14.0 | 82.0 | 15.0 | 83.0 | 20.0 | 81.9 | 18.0 | 116 |
|  |  |  |  |  |  |  |  |  |  |
| Year of birth (10-year interval) | | | | | | | | | |
| 1929 and earlier | 80.0 | 18.0 | 0.0 | - | 0.0 | - | 80.0 | 18.0 | 279 |
| 1930-1939 | 84.0 | 17.0 | 86.0 | 16.3 | 86.0 | 15.8 | 84.5 | 16.7 | 2 707 |
| 1940-1949 | 86.1 | 16.0 | 87.0 | 16.0 | 88.0 | 17.0 | 87.0 | 16.0 | 6 307 |
| 1950-1959 | 86.0 | 16.0 | 88.2 | 16.8 | 90.0 | 15.0 | 87.7 | 15.1 | 11 408 |
| 1960-1969 | 83.5 | 15.7 | 87.0 | 16.0 | 88.1 | 15.0 | 85.8 | 15.0 | 14 150 |
| 1970-1979 | 79.1 | 16.0 | 84.0 | 16.1 | 86.5 | 15.0 | 82.6 | 16.8 | 13 439 |
| 1980-1989 | 72.5 | 14.5 | 79.0 | 18.0 | 84.0 | 16.0 | 77.0 | 18.0 | 14 171 |
| 1990-1997 | 0.0 | - | 73.1 | 16.0 | 77.0 | 16.4 | 76.0 | 16.1 | 4 316 |
|  |  |  |  |  |  |  |  |  |  |
| Locality |  |  |  |  |  |  |  |  |  |
| Rural | 80.0 | 17.3 | 83.5 | 17.1 | 86.0 | 17.2 | 83.0 | 18.0 | 27 636 |
| Urban | 82.0 | 18.0 | 84.0 | 18.0 | 86.0 | 16.0 | 84.0 | 17.0 | 39 141 |
|  |  |  |  |  |  |  |  |  |  |
