# Supplementary material for: Body mass index and waist circumference trajectories across the life course and birth cohorts, 1996–2015 Malaysia: sex and ethnicity matter
Source: Int J Obes (Lond). 2023 Oct 13;47(12):1302–8. doi: 10.1038/s41366-023-01391-5 (PMC10663154; doi:10.1038/s41366-023-01391-5)
Supplement: Supplementary file 10 — Appendix IX [file 41366_2023_1391_MOESM10_ESM.docx]

| **In(waist circumference)** | **Coefficient** | **SE** | **95% CI** | |
| --- | --- | --- | --- | --- |
|  |  |  | **Lower** | **Upper** |
| **Ethnicity** |  |  |  |  |
| Malay | *Reference* | | | |
| Chinese | 0.02 | <0.01 | 0.01 | 0.03 |
| Indian | 0.04 | 0.01 | 0.03 | 0.05 |
| Other bumiputras | <0.01 | <0.01 | -0.01 | 0.01 |
| Others | -0.01 | 0.01 | -0.02 | <0.01 |
|  |  |  |  |  |
| Age (10-year increase) | 0.04 | <0.01 | 0.04 | 0.05 |
|  |  |  |  |  |
| Age^2^ (10-year increase) | <0.01 | <0.01 | <0.01 | <0.01 |
|  |  |  |  |  |
| Cohort (10-year interval) | 0.02 | <0.01 | 0.01 | 0.03 |
|  |  |  |  |  |
| **Ethnic*age** |  |  |  |  |
| Malay | *Reference* | | | |
| Chinese | -0.01 | <0.01 | -0.02 | <0.01 |
| Indian | -0.02 | <0.01 | -0.03 | -0.01 |
| Other bumiputras | <0.01 | <0.01 | -0.01 | 0.01 |
| Others | <0.01 | <0.01 | -0.01 | 0.01 |
|  |  |  |  |  |
| **Ethnic*age^2^** |  |  |  |  |
| Malay | *Reference* | | | |
| Chinese | <0.01 | <0.01 | <0.01 | <0.01 |
| Indian | <0.01 | <0.01 | <0.01 | <0.01 |
| Other bumiputras | <0.01 | <0.01 | <0.01 | <0.01 |
| Others | <0.01 | <0.01 | <0.01 | <0.01 |
|  |  |  |  |  |
| **Ethnic*cohort** |  |  |  |  |
| Malay | *Reference* | | | |
| Chinese | -0.01 | <0.01 | -0.01 | <0.01 |
| Indian | -0.01 | <0.01 | -0.02 | <0.01 |
| Other bumiputras | <0.01 | <0.01 | -0.01 | 0.01 |
| Others | 0.01 | <0.01 | <0.01 | 0.02 |
|  |  |  |  |  |
| Urban | 0.01 | <0.01 | <0.01 | 0.01 |
|  |  |  |  |  |
| **Ethnic*urban** |  |  |  |  |
| Malay | *Reference* | | | |
| Chinese | -0.01 | <0.01 | -0.02 | <0.01 |
| Indian | <0.01 | <0.01 | -0.01 | <0.01 |
| Other bumiputras | <0.01 | <0.01 | <0.01 | 0.01 |
| Others | 0.01 | <0.01 | <0.01 | 0.01 |
|  |  |  |  |  |
| Weight | 0.01 | <0.01 | 0.01 | 0.01 |
|  |  |  |  |  |
| Height | <0.01 | <0.01 | <0.01 | <0.01 |
|  |  |  |  |  |
| Constant | 4.26 | 0.01 | 4.24 | 4.28 |
|  |  |  |  |  |
| **Random-effects** | **Estimate** |  |  |  |
|  |  |  |  |  |
| Period (NHMS) | <0.01 |  |  |  |
|  |  |  |  |  |
| Cohort (5-year interval birth year) | <0.01 |  |  |  |
|  |  |  |  |  |
| State-by-locality | <0.01 |  |  |  |
|  |  |  |  |  |
| Residuals | 0.01 |  |  |  |
|  |  |  |  |  |
| LR test vs. ordinary regression model: chi2(3) = 123.37 Prob > chi2 = <0.001 | | | | |
|  | | | | |

The reference group of the above model is a 40-year-old Malay adult, resided in urban locality and born in 1960
