# Supplementary figures and images for: Body mass index and waist circumference trajectories across the life course and birth cohorts, 1996–2015 Malaysia: sex and ethnicity matter
Source: Int J Obes (Lond). 2023 Oct 13;47(12):1302–8. doi: 10.1038/s41366-023-01391-5 (PMC10663154; doi:10.1038/s41366-023-01391-5)

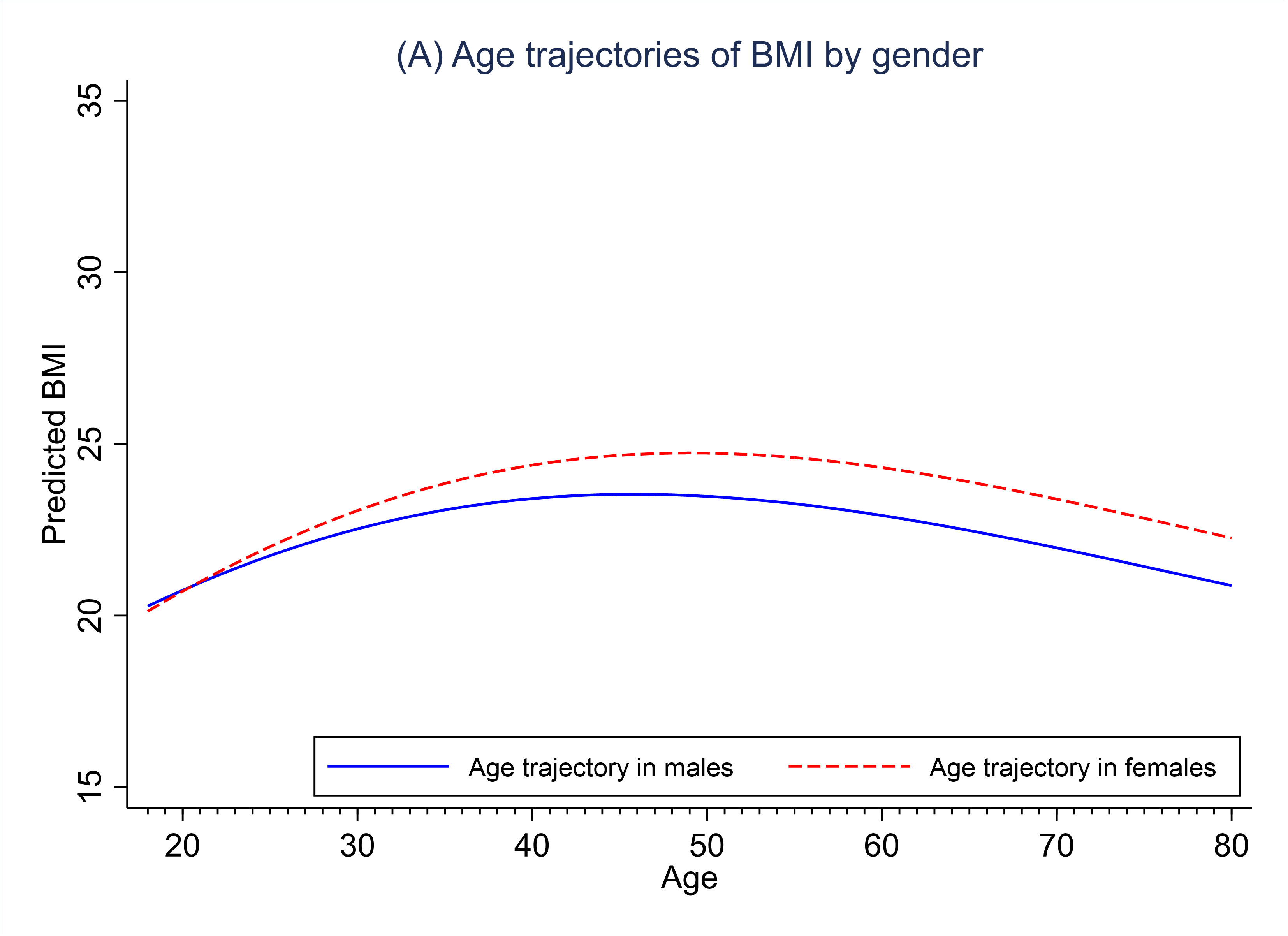

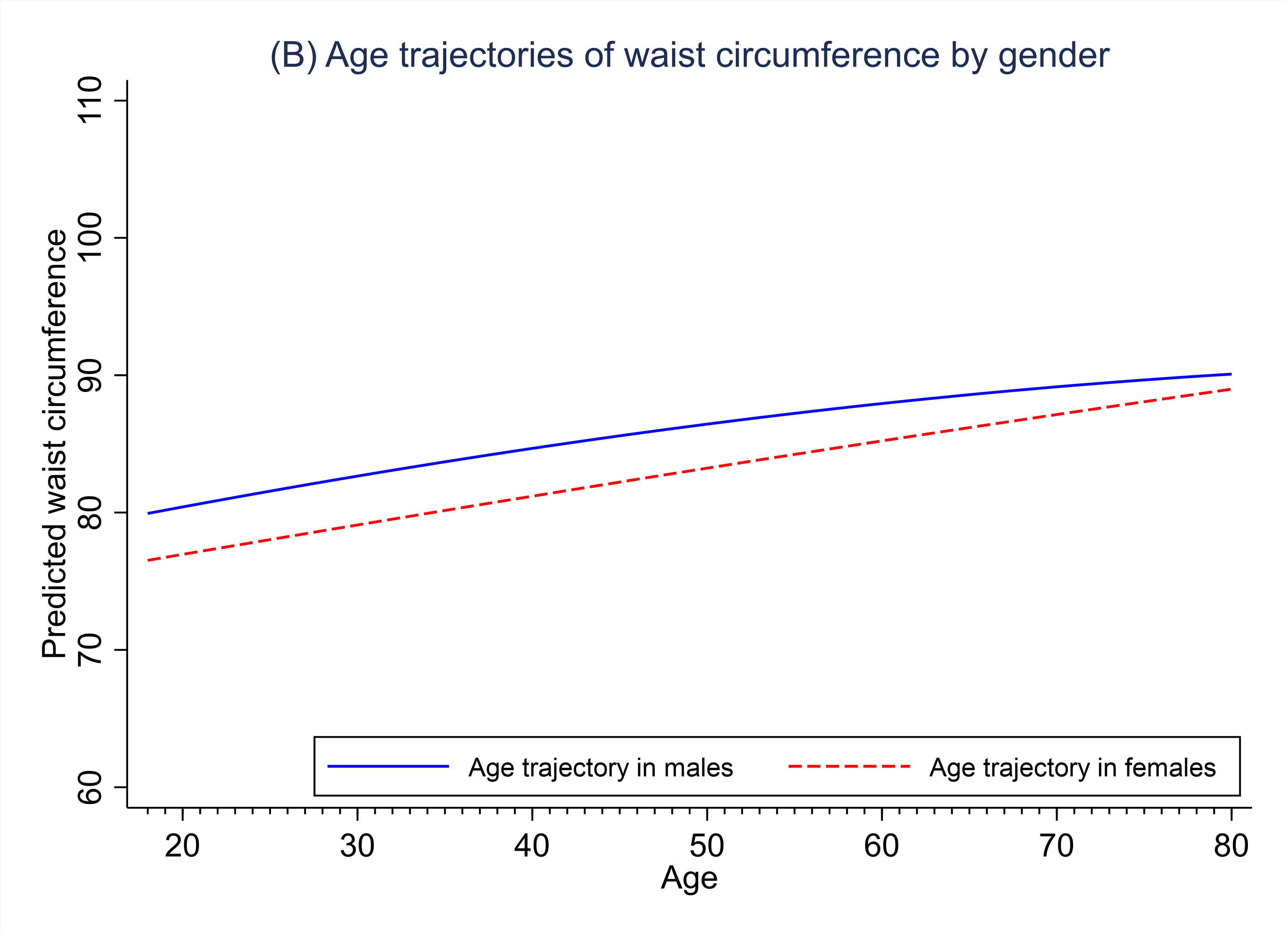

Supplement: Supplementary file 12 — Appendix XI [file 41366_2023_1391_MOESM12_ESM.docx]

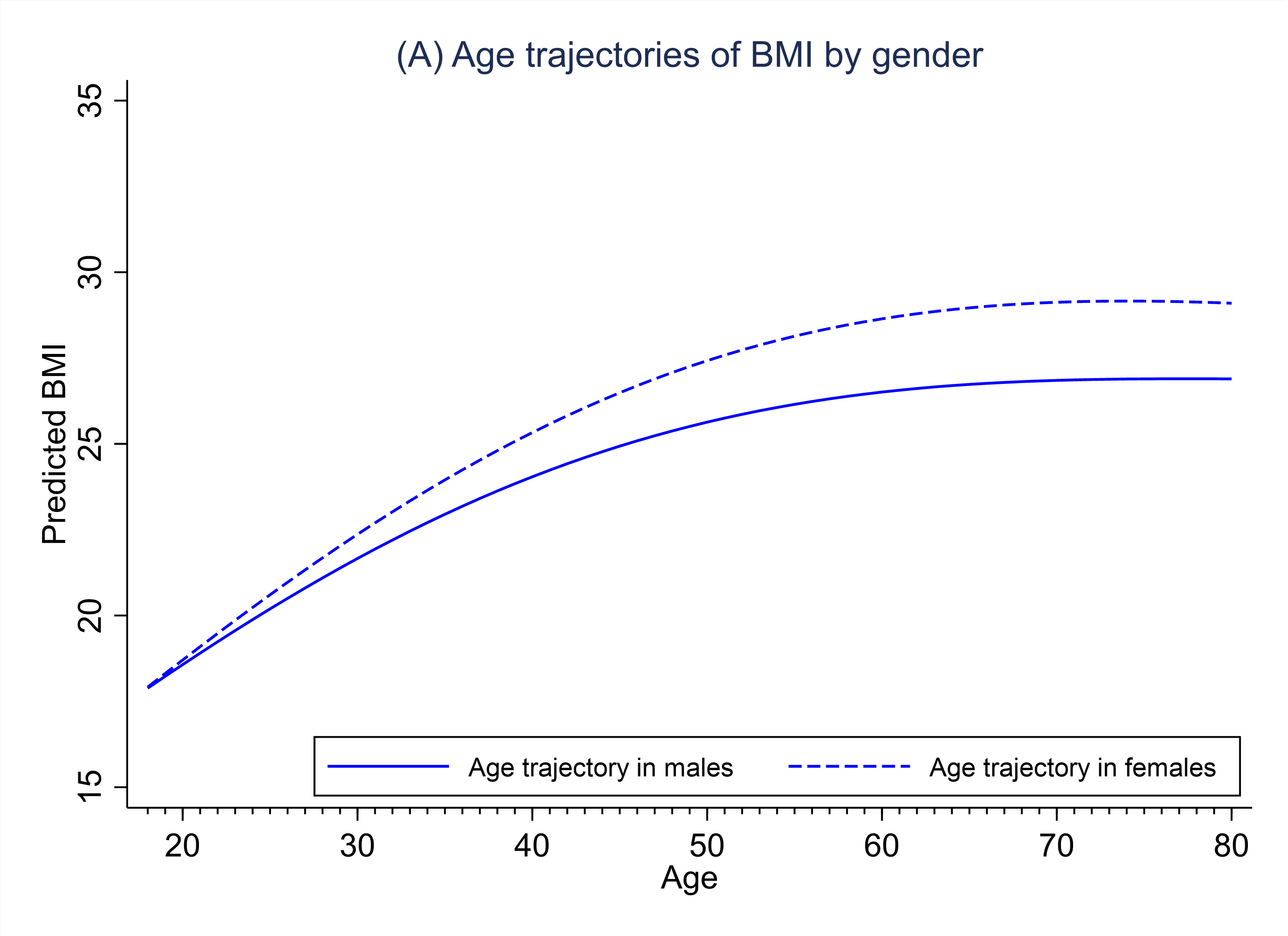

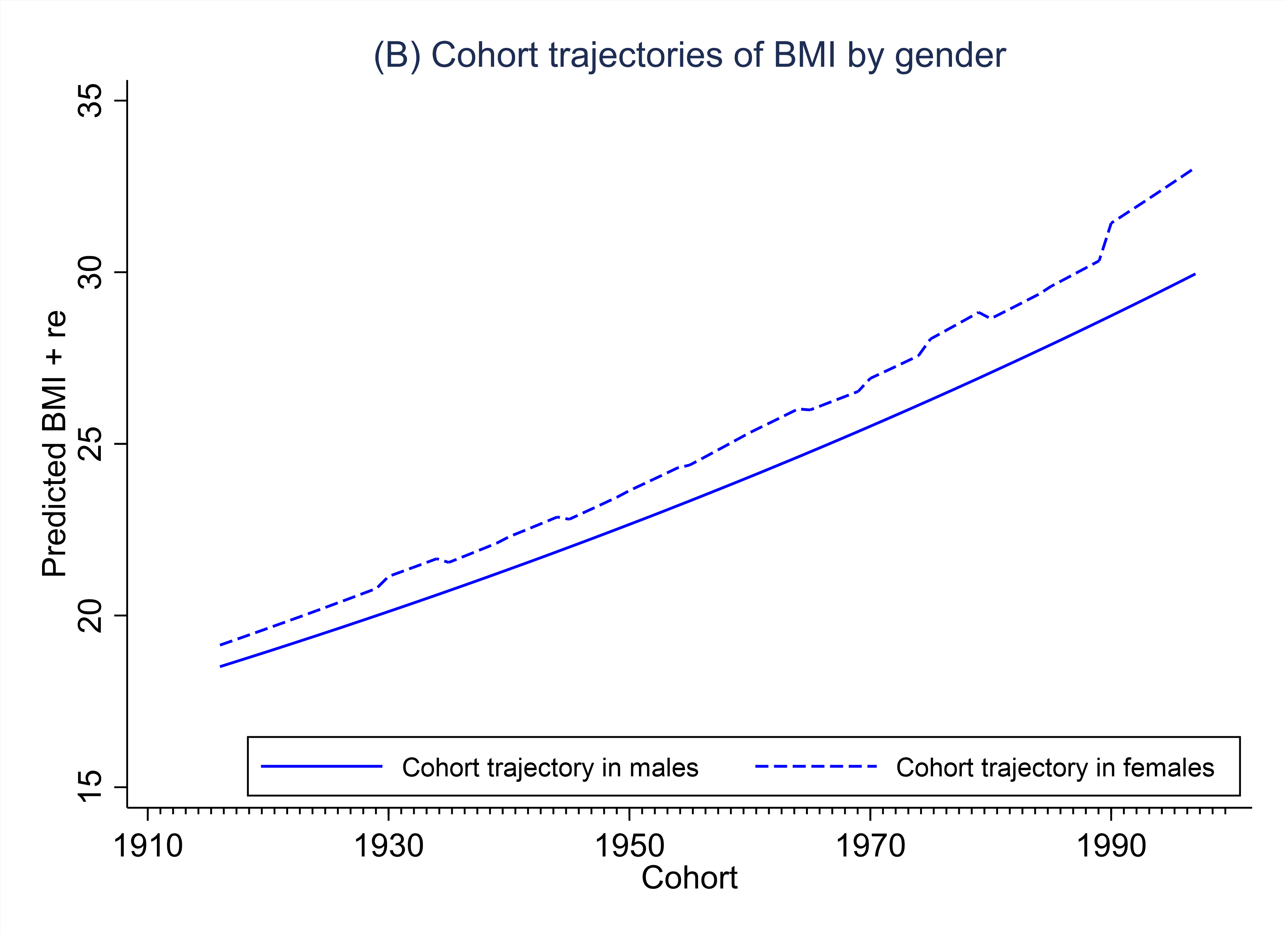


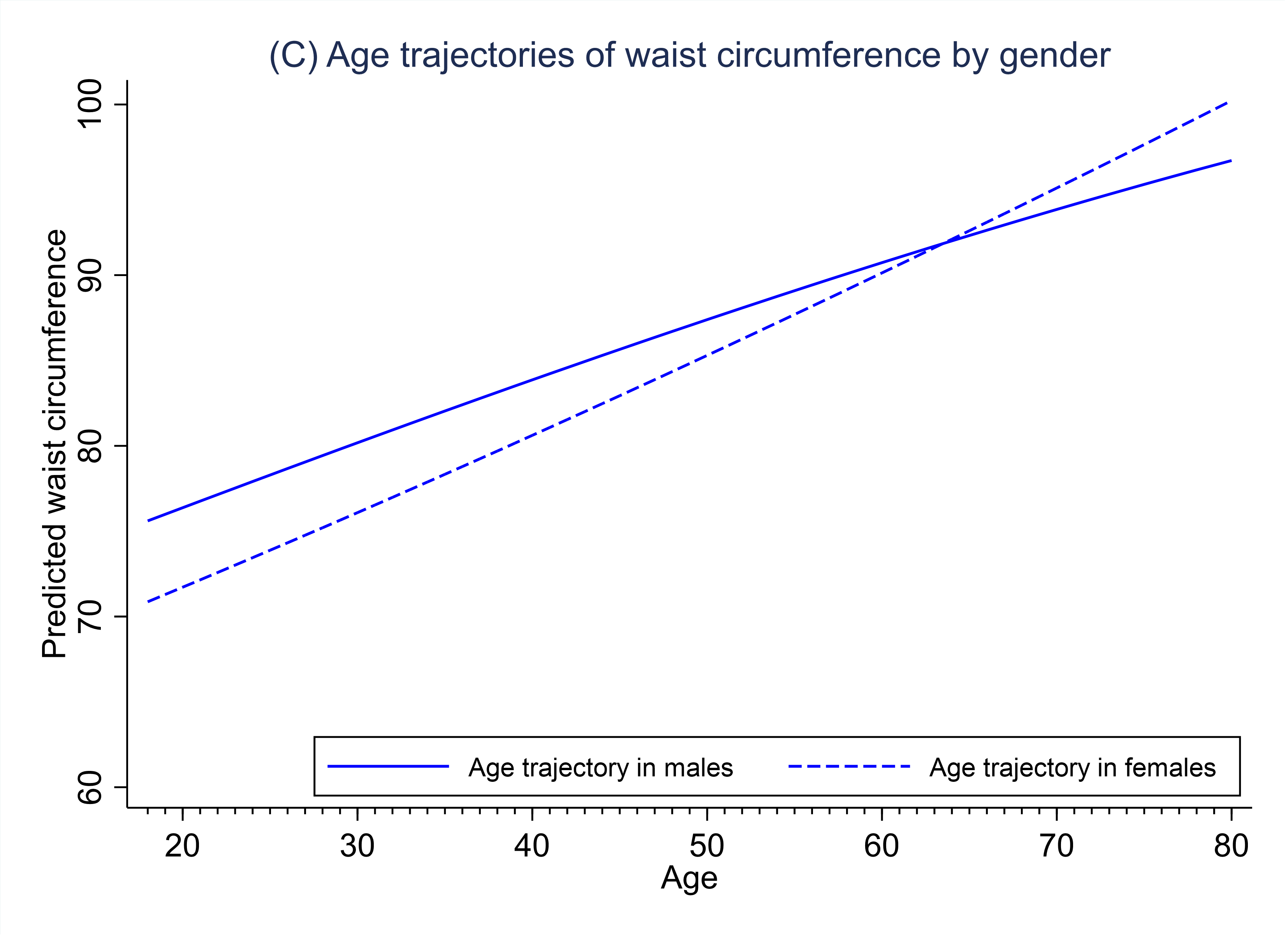

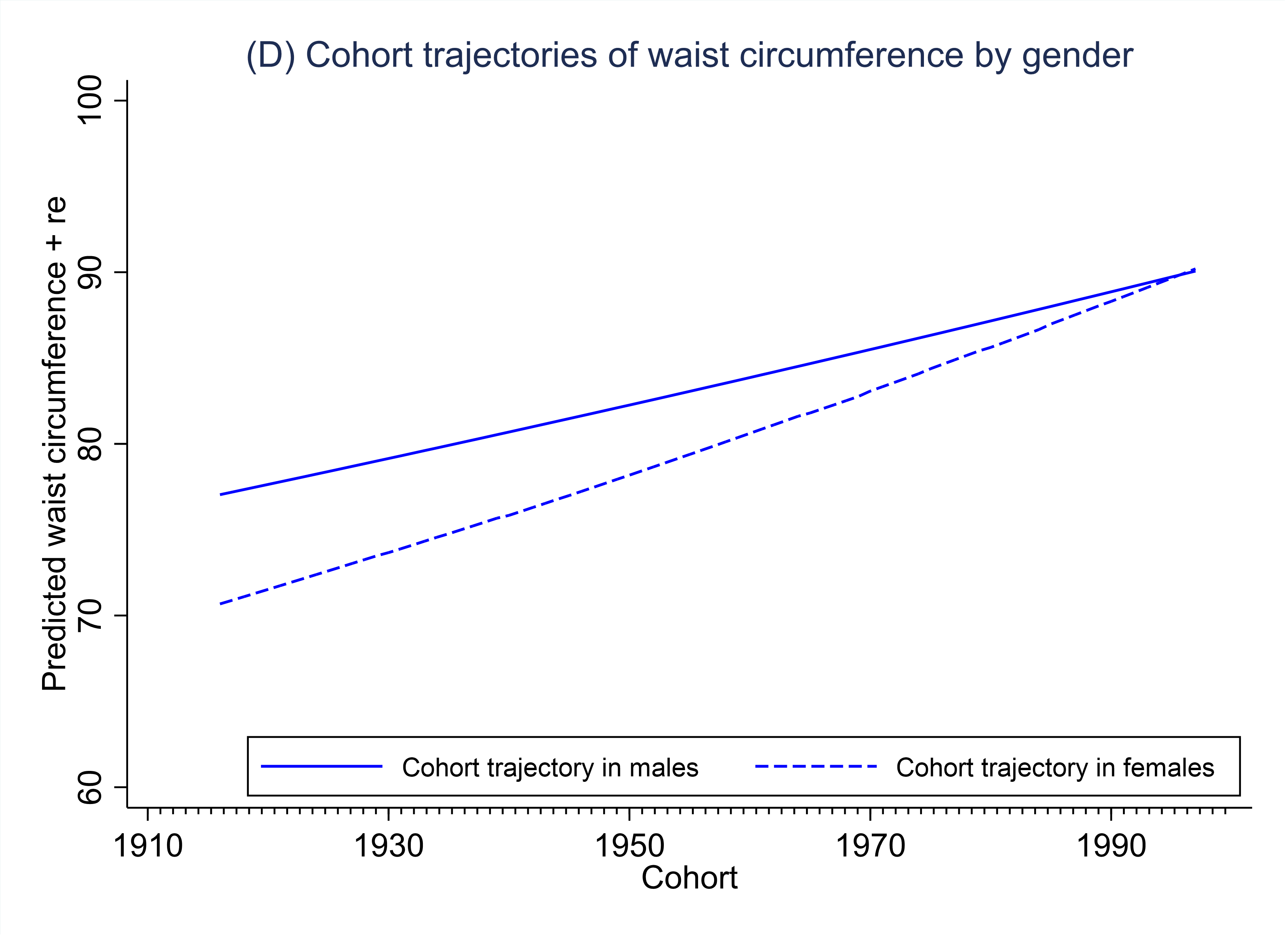

Supplement: Supplementary file 13 — Appendix XII [file 41366_2023_1391_MOESM13_ESM.docx]

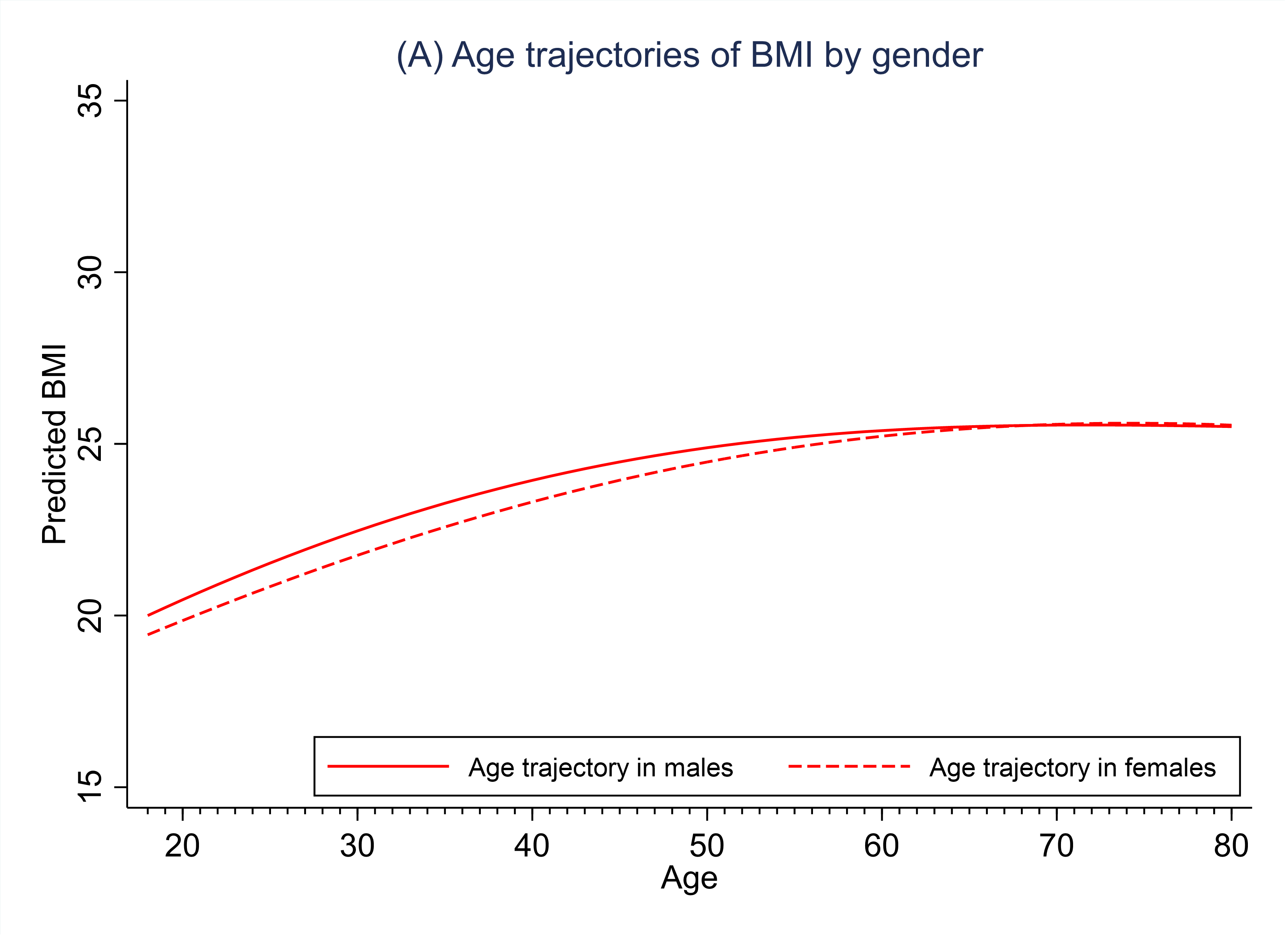

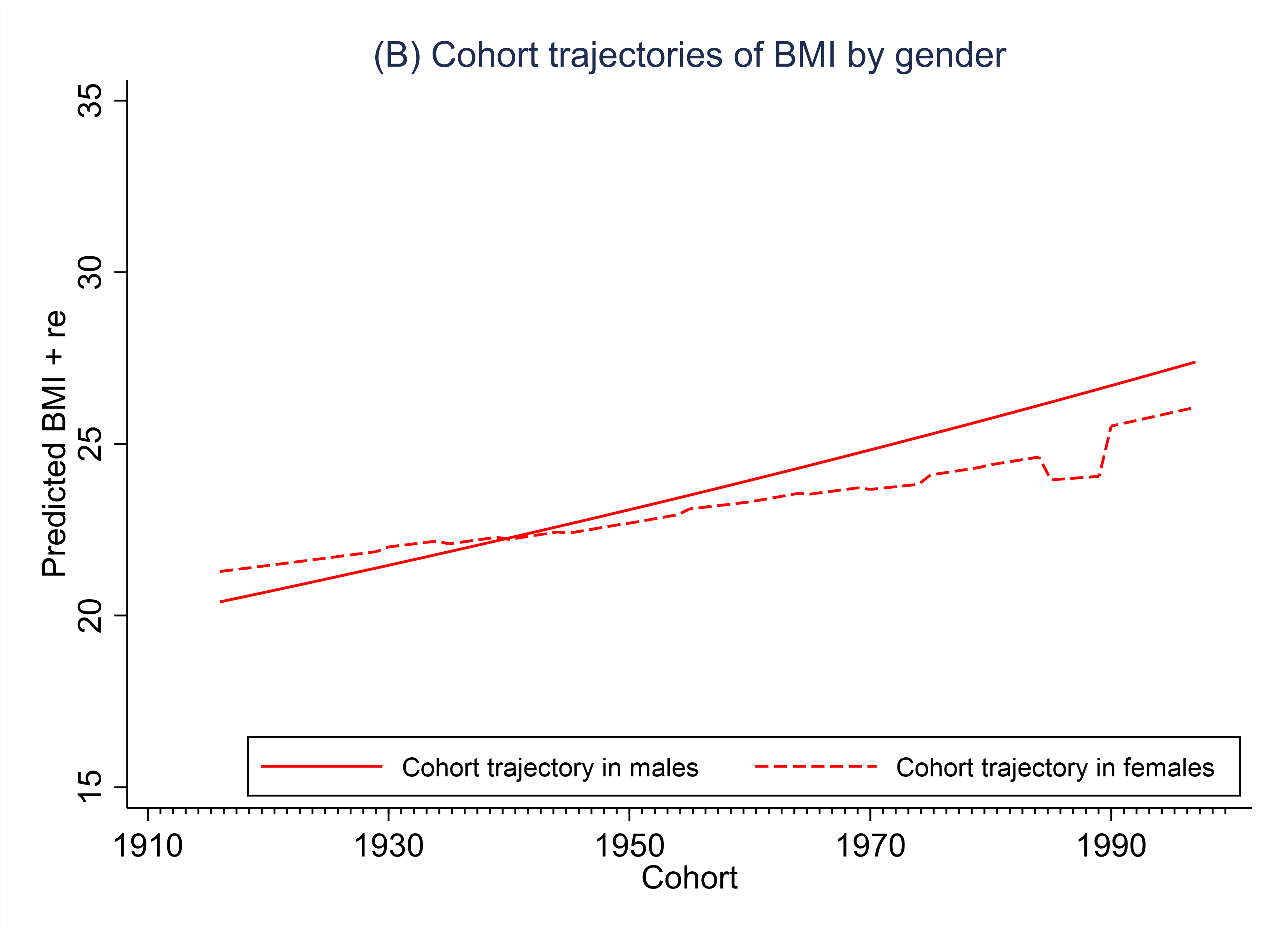


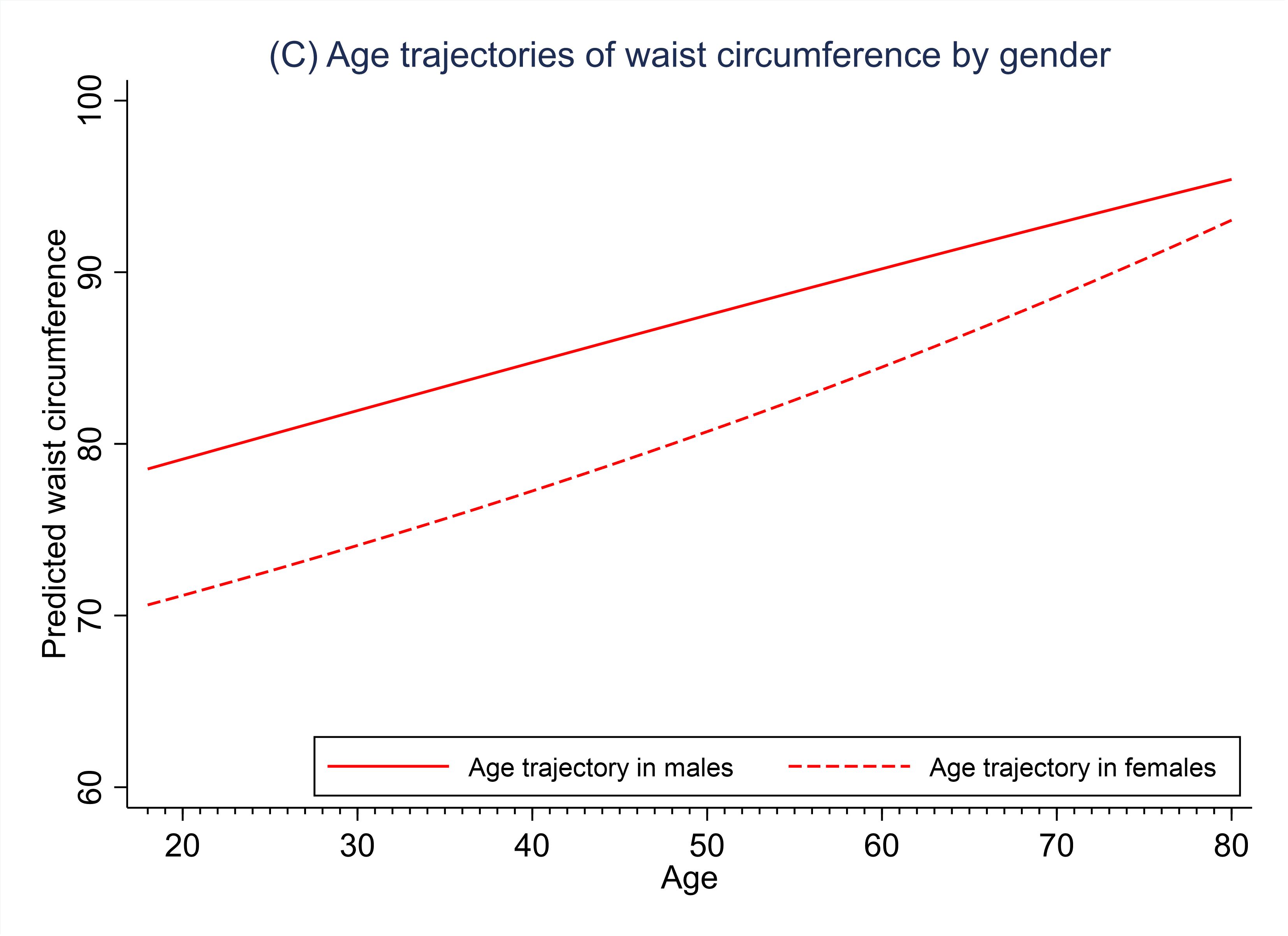

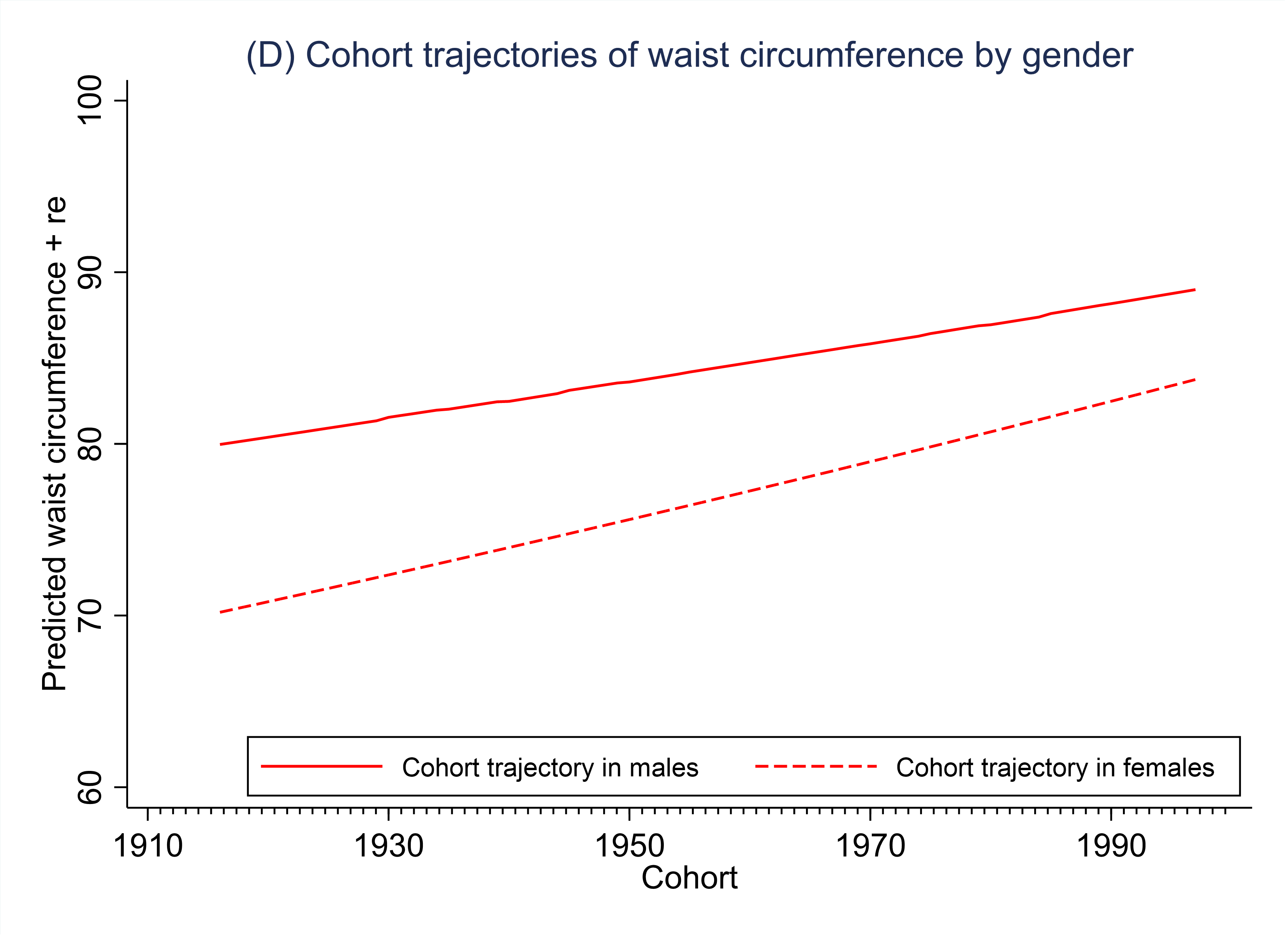

Supplement: Supplementary file 14 — Appendix XIII [file 41366_2023_1391_MOESM14_ESM.docx]
